# Human Cortical Traveling Waves: Dynamical Properties and Correlations with Responses

**DOI:** 10.1371/journal.pone.0038392

**Published:** 2012-06-04

**Authors:** Timothy M. Patten, Christopher J. Rennie, Peter A. Robinson, Pulin Gong

**Affiliations:** 1 School of Physics, University of Sydney, Sydney, New South Wales, Australia; 2 Brain Dynamics Center, Sydney Medical School -Western, University of Sydney, Westmead, New South Wales, Australia; 3 Sydney Medical School, University of Sydney, Sydney, New South Wales, Australia; Cuban Neuroscience Center, Cuba

## Abstract

The spatiotemporal behavior of human EEG oscillations is investigated. Traveling waves in the alpha and theta ranges are found to be common in both prestimulus and poststimulus EEG activity. The dynamical properties of these waves, including their speeds, directions, and durations, are systematically characterized for the first time, and the results show that there are significant changes of prestimulus spontaneous waves in the presence of an external stimulus. Furthermore, the functional relevance of these waves is examined by studying how they are correlated with reaction times on a single trial basis; prestimulus alpha waves traveling in the frontal-to-occipital direction are found to be most correlated to reaction speeds. These findings suggest that propagating waves of brain oscillations might be involved in mediating long-range interactions between widely distributed parts of human cortex.

## Introduction

Since the discovery of neural alpha oscillations with typical frequency of 8 to 12 Hz in normal adult human electroencephalographic activity, neural oscillations with frequencies ranging from 0.05 Hz to 500 Hz have been found to be ubiquitous in both evoked and spontaneous activity [1]. An essential question of these oscillations is how they are spatiotemporally organized, since such organization is believed to be important for the brain, which is a highly distributed system, to coordinate its parts dynamically to give rise to coherent percepts, thoughts, and actions [2]. However, studies of spatiotemporal behaviors of brain oscillations have mostly been limited to their temporal aspects, particularly, their zero-lag synchronization [1]. During the last decades, such synchronization has been extensively studied, with efforts to reveal its potential functional roles in feature binding, memory, and attention [3].

Theoretical modeling studies, including classical studies [4], [5], [6], and modern neural field studies [7]–[12], [13], have suggested that the spatiotemporal behaviors of brain oscillations can organize into propagating waves. In experimental studies, wave patterns have first been observed in electroencephalographic activity [14]. Recently, due to progress in experimental techniques including high-density EEG, multiunit recording, optical imaging and other advanced imaging techniques, a growing number of experimental studies have been showing that propagating waves are widespread in the brain. For instance, such patterns have been found in the olfactory bulb [15], in the visual cortex of various species [16], [17], [18], [19], in the sensorimotor cortex of behaving mice [20], and in the hippocampus [21]. While most of these studies have focused on evoked activity, a few studies have demonstrated the existence of propagating waves in prestimulus spontaneous activity in the absence of external stimuli. Examples include spontaneous wave patterns in visual cortex [17], [22], sensorimotor cortex [23], barrel cortex [24], and human EEG activity [10], [25], [26]. These experimental findings are of particular interest, since they directly demonstrate that instead of being noise, as is conventionally thought, prestimulus spontaneous activity has spatiotemporal structure. Furthermore, this empirical evidence raises several interesting questions: what are quantitative properties of prestimulus spontaneous waves? How are these properties changed in the presence of external stimuli? And more importantly, what are their possible functional roles in brain information processing?

The paper aims to make a step toward answering these questions by investigating the properties of both prestimulus spontaneous waves and poststimulus waves in alpha and theta frequency ranges of human EEG activity, including their speeds, directions and durations. We show that prestimulus spontaneous waves have significant changes in these properties when external stimuli are present, therefore indicating that their collective behaviors change to accommodate the stimuli. Furthermore, based on single-trial analysis of EEG data, we show that the trial-by-trial fluctuations of reaction speeds were significantly correlated to those of prestimulus alpha waves, illustrating that the prestimulus waves have a significant influence on how fast the stimulus is received and processed.

## Materials and Methods

### Experimental paradigm and EEG recordings

The experiment performed by all participants was a typical Go/NoGo paradigm. The subjects sat on a chair and faced a blank black computer screen. The Go stimulus was the presentation of the word PRESS in green on the computer screen while the NoGo stimulus was the presentation of the word PRESS in red. In response to the Go stimuli the subjects were instructed to press a button with their finger, while for the NoGo stimuli the subjects were instructed *not* to press the button. The entire task comprised many individual PRESS stimuli that were grouped into sequences of 6 such that each event in each sequence was the same condition; i.e., either a Go or a NoGo. A set of 20 Go and 5 NoGo sequences were presented in pseudorandom order (a total of 120 Go trials and 30 NoGo trials); we analyzed the Go trials in the study. All PRESS commands were sustained for 500 ms and the speed and accuracy of response were equally stressed in the instructional period before the recordings. The interval between stimulus onsets was 1500 ms. The Go conditions were matched with the corresponding button presses, and the trials where a button was missed were removed.

**Figure 1:**
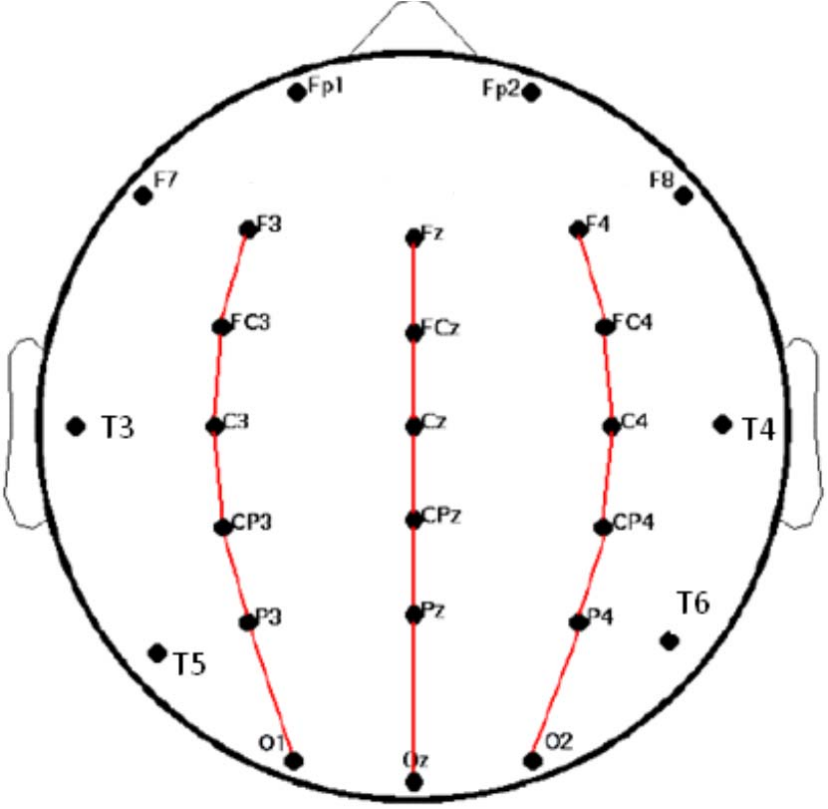
Schematic view of the electrodes placed in accordance with the 10–20 International Systems showing the 26 electrodes. The three chains approximately parallel to the midline are the chains along which we did our traveling wave analysis.

Data collection was done by Brain Resource Ltd (Ultimo, NSW, Australia; www.brainresource.com) and results were made available through the Brain Resource International Database (BRID). Recordings were made at 26 electrode sites from an extension to the International 10–20 System, following previously published methods for acquisition and artifact removal [27]. The layout of the electrodes is shown in Fig. 1. EEG data were recorded at a 500 Hz sampling rate and an A/D precision of 0.06 µV through a NuAmps (Neuroscan) amplifier using an averaged mastoid reference and low-pass third order Butterworth filter with a −6 dB point at 50 Hz. Note that while the main results obtained here were based on the averaged mastoid reference, we have also compared with an average reference, which was obtained by calculating the spatial average of EEG signals at each time moment and subtracting it from all channels, to verify that the results obtained here don't sensitively depend on the reference channel.

Twenty-two subjects participated in the experiments. Prior to the experiment, all had given their written informed consent, and the University of Sydney Human Research Ethics Committee had approved this study. Preprocessing was done as follows: Using a semiautomatic artifact rejection procedure, among the 120 Go trials of each subject we excluded the “bad” trials, in which the absolute voltage difference exceeded 50 µV between 2 neighboring sampling points or the amplitude was outside +100 or −100 µV. The entire recording was rejected if more than 16% of trials were rejected. Six subjects were excluded because of such bad recordings. The remaining subjects (10 males, 6 females, mean age: 32, range: 18 to 56) were healthy, and reported no history of brain injury, disease, or other medical conditions that could influence the normality of the EEG. Also, it is worthwhile to note that, for this age range (18 to 56), EEG characteristics are largely mature and stable; this range avoids both the developmental changes in childhood and the changes due to old age [28], [29], [30]. For the remaining 16 subjects, we found that the number of bad trials varied between the different subjects, ranging from 5 to 19 trials. For inter-subject consistency in our analysis, we therefore used 100 clean trials from each subject's dataset.

### Data analysis

The time-frequency information and phases of the EEG data were computed using the continuous wavelet transform with complex Morlet wavelet. In contrast to standard FFT analysis using fixed time windows, the wavelet analysis uses longer time windows for lower frequencies than for higher ones. Suppose that 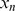 is the sampled signal from one channel, then its wavelet transform is
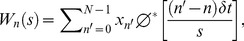
(1)where *n* is the time index, asterisk indicates the complex conjugate, 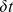 is the sampling time interval of the signal 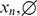 is the wavelet function with *s* being the scale of the function. The wavelet function 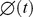 is
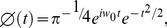
(2)Where *w_0_* is the nondimensional frequency, here taken *w_0_* = 6 to satisfy the admissibility condition of wavelet [31]. Since *W_n_* (S) is a complex number, it can be represented as 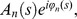 in which *A_n_* is the amplitude and 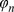 is the phase, with
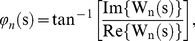
(3)Where 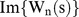 is the imaginary part of 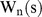 and 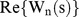 is its real part. Equations (1)–(3) enable each voltage time series to be transformed into time series of phase (modulo 2π).

**Figure 2:**
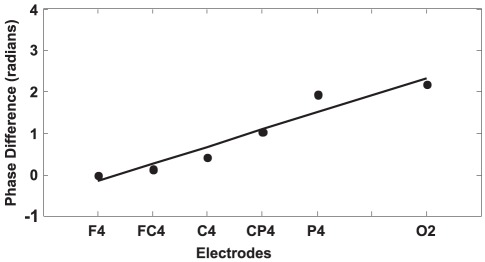
An illustration of gradual phase shift along the chain of electrodes from F4 to O2 for one typical subject. The dots for phase difference values and the solid line for a linear fit to them.

EEG records potentials on the scalp, and it has low pass filter effect on brain signal; this effect prevents the detection of high temporal frequency waves with low wavelengths. In other words, EEG signals are more suitable to “see” global waves with the longest wavelength. In the present study, such large-scale global waves traveling either in the frontal-to-occipital (F-to-O) or occipital-to-frontal (O-to-F) direction were mainly studied, because the electrodes available to us were sparser in other directions. Nevertheless, it is worth noting that other studies have argued that the global waves propagating in the two directions are more prevalent than in other directions, since corticocortical fibers are significantly more than commissural and thalamocortical ones [10]. Our analysis focused on large-scale waves traveling parallel to the midline along the three chains shown in Fig. 1. To detect the traveling waves, we investigated how the phases of oscillations were organized over the selected electrodes. Relative phases, as a function of time, were calculated for each of the electrodes in the three chains shown in Fig. 1. Specifically, a phase difference at sampling instant *n* and frequency scale *s* was calculated using
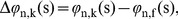
(4)where *k* denotes an electrode in a given chain and *f* denotes the frontmost electrode of the chain (F4 for the right chain, Fz for the midline, and F3 for the left one). The calculations were performed for each electrode in the chain and in the order of their occurrence starting from the front and moving to the back. The existence of waves was then analyzed by investigating these phase differences. If they increased or decreased progressively as the distance to the reference channel increased, the activity pattern was regarded as propagation of a traveling wave. For instance, Fig. 2 illustrates such a case with a progressive phase shift along the right chain shown in Fig. 1 at time t = 121.83 s. As indicated in this figure, as the distance to the reference channel F4 increased, the phase difference with respect to that channel progressively increased. The black line shown in Fig. 2 is the linear best fit to the phase differences.

To test whether the slope of the linear fit is significantly different from zero, phase values along the chain were randomly shuffled, and then a linear fit was applied to them to get a slope value. This randomization procedure was repeated for 1000 times to yield a distribution for the significant test; the original slope was counted to be significantly different from zero, if it exceeded the 95^th^ percentile of the distribution. When the slope was significantly different from zero, its sign was then used to determine the wave's direction: a positive slope value indicates a wave traveling in the F-to-O direction, while a negative one indicates a wave traveling in the opposite direction. The large-scale traveling waves parallel to the midline were detected by matching the phase shifts from the three chains of electrodes; that is, if each of the regions had a progressive shift in phases and it progressed in the same direction, then this constituted such a large scale wave.

The large-scale waves along the electrode chains may continue for some time intervals, meaning that during these intervals the phase difference 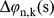 kept nearly constant. In other words, during these intervals, the waves are manifest in phase locking activity over the electrode chains. In order to obtain these intervals, we used the single-trial phase locking value measured across successive time steps [31], [32],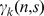, which is defined below
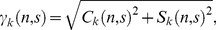
(5)where
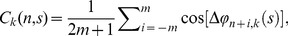
(6)

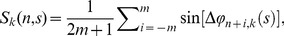
(7)where *n* is time index, and *s is* the frequency scale, 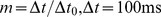 as used in [25], [33], and 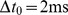 (the sampling interval of the EEG signal). We then got the average value of the phase locking values:
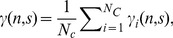
(8)Where 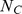 is the number of electrode pairs (the pairs used in Eq. 4), 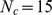 in our current study. For 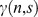 defined above, its values range from 0 to 1, with 1 indicating the strongest phase locking. If time *n* is within the range 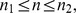, during which 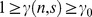 (with 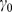 close to 1.0), 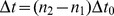 then is the duration of the global propagating waves propagating either in the F-to-O or the O-to-F direction. 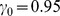 was used in the current study. We, however, found that the use of other values close to 1.0 would not change the durations of waves significantly; this is similar to other studies [25].

The large scale waves may arise from some trivial causes such as the layout of electrodes, or methodological processes, such as the wavelet based calculation of phases. To test the null hypothesis that the existence of large-scale waves is due to such trivial effects, we also repeated the analysis on surrogate data that was constructed by Fourier-based randomization of the original EEG data [34], which were generated by preserving the mean power spectrum of the original data while randomizing the phase of the various frequency components. Two hundred such surrogate datasets were computed for each trial and then the same procedure was repeated on them to get 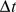, as for the original EEG data, therefore resulting in a null distribution of durations. The statistically significant durations of waves in the original EEG data were those durations that exceeded the 95th percentile of the null distribution of 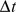, and these waves were regarded as statistically significant wave events, which were used for our further analysis.

We considered the 500 ms preceding stimulus onset and the 500 ms time epoch after that. For each 500 ms epoch, we calculated the total wave duration in the whole trial,
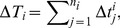
(9)where *i* denotes the *i*th trial of a subject, and 
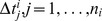
 is the duration of the *j*th significant traveling wave event (as defined above), and 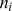 denotes the total number of such wave events in the trial. If among the total 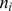 significant wave events during 500 ms time interval before the stimulus onset, there are 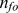 such events traveling in the frontal-to-occipital direction, then its percentage is 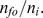 Also, we calculated the distributions of waves in occipital-to-frontal direction 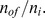 The speed of each statistically significant wave event was then calculated by averaging the slopes of the linear fit lines of the phase shifts along the three chains (Fig. 1), and the speed of the waves of a whole trial was obtained by averaging the speeds of the wave events occurring in the trial.

To evaluate whether there were significant changes in the properties of waves such as their directions and durations for prestimulus and poststimulus 500 ms epochs, a randomization test [35], based on 5000 permutation runs over the epochs, was carried out. If the *p*-value of this randomization test was below 0.05, then wave properties were regarded to have a significant difference between the two epochs.

### Correlation between waves and reaction rate

To test for a potential functional role of traveling waves, we studied how their properties would be correlated with subject performance such as reaction speeds. The reaction time of each of the 100 Go trials was measured from the stimulus onset to the time point when the subjects pressed the button. Regarding the corresponding EEG data, we calculated 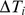 (Eq.9) for 500 ms epoch before and after stimulus onset. The fraction within each 500 ms epoch is defined to be 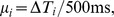 which was calculated for all 100 trials for individual subjects. For each subject, we obtained its maximal fraction μ_max_. To make the fractional values comparable over different trials of individual subject, they were rescaled to a number between 0 and 1 by dividing them by μ_max_. Hence, this established a wave index 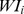 for the *i*th trial
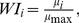
(10)where *i* = 1,…,100 are the trials. A wave index value close to 0 indicates waves with relatively small duration, while a value close to 1 indicates the existence of waves with relatively large duration for a particular subject.

Similarly, a reaction speed index, *RSI,* was established to scale the reaction speed in each trial to a number between 0 and 1. This was done by first taking the reciprocal of each trial's response time (*r*) to give a response speed *ν = 1/r*, such that the larger the value ν, the faster the response. The reaction speed index of each subject was then calculated by dividing all speeds by the largest speed across all 100 trials of the subject,
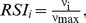
(11)Where 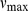 is the maximal speed among the 100 trials for a given subject. This established the reaction speed index, where a value close to 0 indicates a relatively slow response and a value close to 1 indicates a relatively fast response.

We then calculated the Pearson correlation coefficient between the trial-by-trial variations in the response speeds and those in the wave:
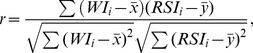
(12)Where 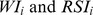 are the wave and reaction speed indices of the *i*th trial, and 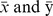 are their corresponding mean values over the 100 trials. We used a permutation test method [35] to test the significance of the correlation. In our study, this procedure involved randomly exchanging the wave indices across trials, and it was repeated 5000 times for each subject to yield a distribution for a significance test. For each subject, an original correlation was counted as significant if it exceeded the 95^th^ percentile of the distribution of permuted values.

## Results

### Time-frequency analysis

**Figure 3:**
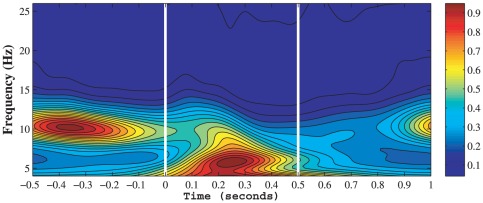
Wavelet power spectrum of a typical subject averaged over all trials and electrodes in normalized power units. The stimulus onset time is at 0 ms.

To detect the main oscillatory frequency bands of EEG activity, on which more fine-grained analyses were performed, we first carried out a time-frequency analysis for the EEG signals. We studied power spectrum from 0.5 to 80 Hz, and found that the signals showed very little power above 26 Hz compared to that of lower frequencies. Thus, 26 Hz was established as the upper cut off frequency for further investigation. We also closely inspected the delta range (0.5–4 Hz) oscillations and found that the Go and NoGo conditions had indistuiguishable characteristics, indicating that the delta oscillations could be neglected in the comparison between the two conditions. Therefore, 4 Hz was established as the lower cut off frequency in our study. A wavelet transform from 4–26 Hz was then performed on the entire time series of each subject. To better present the dominant frequencies of EEG activity, for each subject the wavelet power spectrum was averaged over all 100 Go trials and then averaged over all electrodes. We found that most of the subjects had strong alpha oscillations (8–12 Hz) before the stimulus onset, and strong theta oscillations (4–8 Hz) after the stimulus onset; representative results from a subject are shown in Fig. 3, from which we can see that such alpha oscillations peaking at 10.6 Hz and theta oscillations peaking at 5.8 Hz.

### Traveling waves

**Figure 4:**
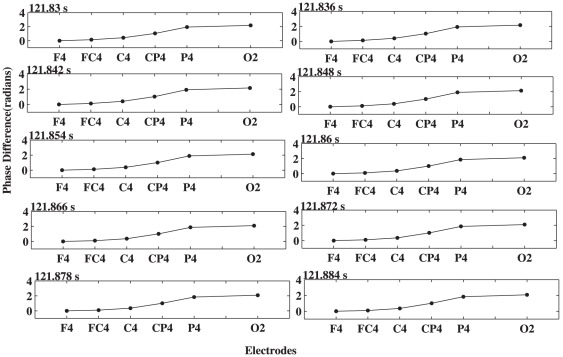
Phase differences along the chain of electrodes, including F4, FC4, C4, CP4, P4, and O2, at different times with step size of 6 ms.

**Figure 5:**
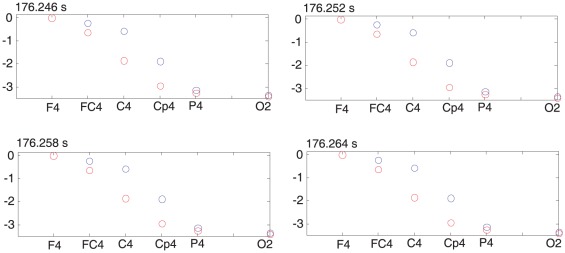
Phase differences along the chain of electrodes, including F4, FC4, C4, CP4, P4, and O2, at different times with step size of 6 ms. Red circles are the calculated phases for EEG signals with the average mastoid reference, black ones for EEG signals with an average reference; this result indicates that these phase differences do not sensitively depend on the reference channel.

To calculate phases for the prominent alpha and theta oscillations, we adjusted the scale of the wavelet to make it centered at the peaks of these oscillations accordingly, which were determined individually on the basis of the above time-frequency analysis. After obtaining the phases of these oscillations, we then calculated phase differences for the electrodes along each of the three chains shown in Fig. 1. We found that there were significant time intervals during which there were progressive phase shifts along these chains. Figure 4 shows such a typical example for electrodes along the F4 to O2 chain with phase shifts in F-to-O direction, and Fig. 5 for phase shifts in O-to-F direction. The phase shift over the electrodes was then fitted to get a straight line.

**Figure 6:**
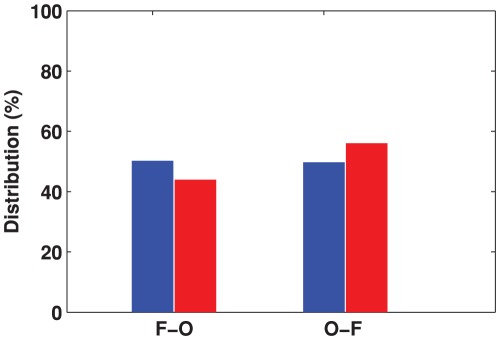
Distribution of alpha waves between the two directions (frontal-to-occipital direction and occipital-to-frontal direction) before and after stimulus onset. The blue bars indicate the prestimulus waves measured in the 500 ms interval before stimulus onset and the red bars indicate the poststimulus waves measured in the 500 ms interval after stimulus onset.

First, the direction of traveling waves was investigated. Figure 6 shows the percentage of alpha waves in each direction, which was calculated by averaging 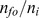 and 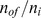 (see Methods) across trials and subjects. Similarly, the distribution of waves during post-stimulus 500 epochs was calculated. As shown in the figure, during the 500 ms time interval before the stimulus onset, the frontal-to-occipital and occipital-to-frontal propagation directions of the spontaneous waves were roughly evenly divided at 50.3±2.2% and 49.7±1.8%, and there was no significant difference between them (permutation test, *p*>0.05). However, after the stimulus onset the occipital-to-frontal waves became more common (56.0±1.9% compared with the frontal-to-occipital direction at 44.0±2.0%). The change in the propagation direction was significant (*p*<0.005), based on the permutation test (see Methods). This indicates that the external stimuli evoke more waves traveling in the O-to-F direction than the F-to-O direction.

**Figure 7:**
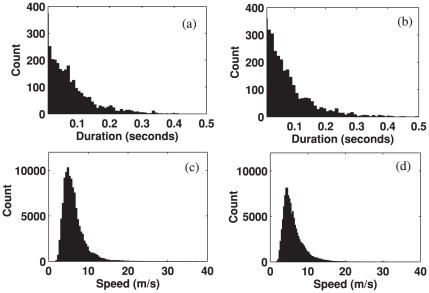
Histogram of the durations and speeds of alpha traveling waves during 500 ms time intervals before and after external stimulus onset, which were measured over all subjects. (a) Durations of prestimulus waves. (b) Durations of poststimulus waves. (c) Speeds of prestimulus waves. (d) Speeds of the poststimulus waves.

The histograms of the durations of the alpha waves before and after the stimuli onset are shown in Figs. 7(a) and 7(b). The average of durations and that of speeds across trials and subjects are summarized in Table 1. The average duration of the prestimulus spontaneous waves was 73 ms (SD = 15 ms) while that of poststimulus waves was 62 ms (SD = 11 ms) (Table 1), which was a reduction of 13% (permutation test, p<0.01). Thus, it appears that the average duration of alpha waves also experienced a collective change due to stimuli. Figures 7 (c) and 7(d) show histograms of the speeds, which were mostly concentrated between 2 m/s and 15 m/s. The average speed of the prestimulus alpha waves was 6.5 m/s (SD = 0.9 m/s), and that of the poststimulus alpha waves was 6.2 m/s (SD = 0.9 m/s) (as summarized in Table 1); there were no significant changes in the speeds of alpha waves due to stimuli (permutation test, p>0.05).

**Table 1:** Average durations and speeds of pre- and post stimulus alpha and theta oscillations.

wave types	prestimulus alpha	poststimulus alpha	prestimulus theta	poststimulus theta
duration (ms)	73±15	62±11	84±12	112±18
*f*	15%	12%	17%	22%
speed (m/s)	6.5±0.9	6.2±0.9	4.0±0.6	4.0±0.8

The fraction of time for traveling waves during the pre- and post-stimulus 500 ms is 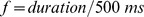.

**Figure 8:**
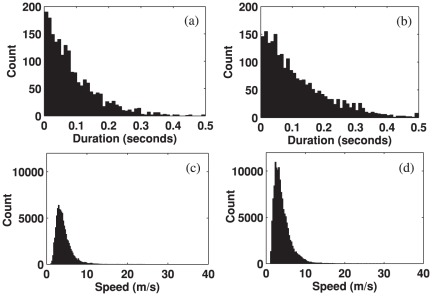
Histograms of the durations and speeds of prestimulus and poststimulus theta waves measured for all subjects. (a) Durations of prestimulus waves. (b) Durations of poststimulus waves. (c) Speeds of prestimulus waves. (d) Speeds of poststimulus waves.

A similar analysis was then performed for the theta waves. Figure 8 shows histograms of the average durations and speeds of the theta waves for all trials and subjects during the epochs, 500 ms before and 500 ms after stimulus onset. As summarized in Table 1, the average durations of the prestimulus and poststimulus theta waves across trials and subjects were 84 ms (SD = 12 ms) and 112 ms (SD = 18 ms) respectively, and the difference between the two intervals was significant (permutation test, p<0.001). The theta waves significantly increased in average duration, unlike the alpha waves, whose duration decreased. Similarly, the histograms of the calculated speeds are shown in Figs. 8 (c) and (d). The average speeds for the spontaneous and poststimulus theta waves were 4.0 m/s (SD = 0.6 m/s) and 4.0 m/s (SD = 0.8 m/s) respectively, lower than the alpha speeds. There were no significant changes in the speed during the two intervals (permutation test, p>0.05).

### Correlation between waves and reaction rate

**Figure 9:**
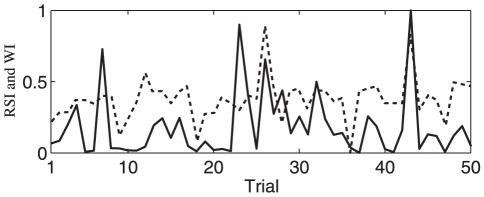
The reaction speed index (*RSI*) and wave index (*WI*) over trials for a typical subject; solid line for *RSI* **and dashed line for** ***WI*** **.** The wave index was calculated for the F-to-O alpha waves.

Figure 9 shows the typical wave index of the prestimulus alpha waves and reaction speed index of one subject, as defined above, over trials. It is well known that reaction time fluctuates significantly over trials even when external stimuli are identical. For all subjects in our recordings, one can clearly see such variations. As shown in Fig. 9, there were great trial-by-trial fluctuations in both indices. However, the two indices appear to follow a similar fluctuating trend; larger wave occurrences have larger response speeds (i.e., subjects reacted faster), and smaller wave occurrences have smaller response speeds (i.e., subjects responded slower). This, therefore, demonstrates a potential correlation between the two indices. To directly quantify this and to test whether the trial-by-trial fluctuations of the waves can predict the response speeds, we calculated the Pearson correlation coefficient of the two indices (see Methods) for the 16 subjects. The results for the alpha waves during 500 ms preceding stimulus onset, including correlation values and the corresponding *p* values obtained from permutation tests (see Methods), are summarized in Table 2; the results indicate that prestimulus alpha waves had significant correlations with reaction speeds (*r = 0.58, p = 0.001*).

**Table 2:** Correlation coefficient (*r*) and corresponding *p* values between the wave index and reaction speed index for pre- and post-stimulus alpha and theta waves traveling in F-to-O and O-to-F directions.

prestimulus alpha wave		poststimulus alpha wave		prestimulus theta wave		poststimulus theta wave	
F-to-O	O-to-F	F-to-O	O-to-F	F-to-O	O-to-F	F-to-O	O-to-F
r = 0.55 *p* = 0.002	r = 0.1 *p* = 0.6	r = 0.1 *p* = 0.6	r = 0.05 *p* = 0.9	r = 0.05 *p* = 0.8	r = 0.08 *p* = 0.6	r = 0.04 *p* = 0.8	r = 0.06 *p* = 0.7

We next tested whether the direction of the traveling alpha waves could affect subjects' responses. To do this, the prestimulus waves traveling in the two directions (the F-to-O direction and the O-to-F direction) were analyzed separately and were found to have different relations with the response speeds. As shown in Table 2, prestimulus F-to-O alpha waves had significant correlations with reaction speeds (*r = 0.55, p = 0.002*), but there were no significant correlations between O-to-F waves and reaction speeds. To investigate whether the prestimulus and poststimulus alpha waves related differently to response speeds, we did the same correlation analysis for wave and reaction speed indices for the 500 ms epoch after stimulus onset. We found that there were no significant correlations between poststimulus alpha waves and reaction speeds (Table 2). To determine whether the prestimulus and poststimulus theta waves were related to subject's performance, we therefore repeated the above trial-by-trial correlation analysis to these wave activities, but we found no significant correlations between them and reaction speeds (Table 2).

## Discussion

We have systematically quantified the directions, speeds, and durations of traveling waves in both alpha and theta frequency bands. Our results indicate that, on average, the alpha waves were faster than the theta waves:6.5±0.9 m/s for the alpha waves and 4.0±0.9 m/s for the theta wave, with a ratio of 1.6±0.5 between them. Our calculated speeds of alpha waves are similar to the speeds measured with human EEG signals of 3.6–10.4 m/s [36], 7–11 m/s [16], or 3–8 m/s [37]; these data agree closely with the estimated peak in the distribution of myelinated corticocortical propagation speeds of roughly 6–9 m/sec [10], [38]. Thus, our results together with others [16], [36], [37] suggest that the important properties of EEG large-scale wave dynamics appear to depend on corticocortical fibers. This interpretation, however, does not rule out the possibility that the thalamus may exert an important influence on cortico-cortical propagation via thalamocortical reentrant loops. As far as we are aware of, there are no other published results about the speed of theta waves for human subjects. Hence, no comparison can be done at this stage. It is also interesting to note that the propagating waves of higher frequency are faster than those of smaller frequency, is generally consistent with a modeling study [39], in which it has shown that cortical wave speeds should increase as the frequency increases up until the alpha peak. In addition, the ratio between alpha wave speed and theta wave speed, which was 1.9 calculated based on one example for typical model parameters [39], is within the range of 1.6±0.5 as found in the current study.

Although propagating waves have been found to be common in both prestimulus spontaneous and poststimulus activity, there have been very few studies that investigated the changes of spontaneous traveling wave properties due to an external input. For instance, there was a brief examination of the change of wave velocities for wave activity with a frequency range of 0.5–4 Hz in anesthetized rat visual cortex [17]. In contrast, we have done systemic studies on such changes; our results clearly demonstrate that there were several interesting changes for the spontaneous waves when external stimuli were present. First, there were statistically significant changes in the durations of both alpha and theta waves; from the prestimulus 500 ms epoch to the poststimulus 500 ms epoch, the average durations changed from 73 ms to 62 ms, and from 84 ms to 112 ms, for the alpha wave and the theta wave respectively. Another compelling finding was that of the changes to the distributions between directions of the waves; the alpha waves without the stimuli traveled almost equally in both directions, but following the stimuli the waves became more concentrated in the direction from the occipital to frontal areas. These results suggest that when external stimuli were present, propagating waves are modulated and triggered by external stimulus [40]. Also, it is worthwhile to note that in our study the stimulus-related changes differed for the two frequency ranges. The dissimilarities between theta and alpha waves indicate that they might have different roles at the different stages of brain information processing; indeed, it has been shown that among a range of its functional roles, alpha activity is more related to internal cognitive expectation, and theta activity is more associated with sensation and behavior monitoring [41]. Thus, the decrease and increase of alpha and theta wave direction, respectively, might indicate a reduction of cognitive expectation and an increase of behavior monitoring after the external stimulus.

We also performed correlation analysis between traveling waves and reaction speeds on a single trial basis, and both prestimulus spontaneous and poststimulus waves were studied. By far the most significant relationship with reaction speeds was found with the prestimulus alpha waves. Furthermore, we found that the prestimulus alpha waves propagating in the frontal-to-occipital direction had a correlation with reaction speed, but the waves propagating in the opposite direction didn't have such correlation. Our results accord well with a growing number of recent studies that have shown that prestimulus brain oscillations are correlated with various perceptual and cognitive functions, such as visual perception performance [42], [43], [44], the speeds of responses [45], and perception of ambiguous audiovisual stimulus [46]. However, rather than examining the amplitude or the degree of synchronization of prestimulus brain oscillations as in the previous studies, the current study focuses on the functional relevance of these oscillations on the basis of their spatiotemporal behavior in terms of waves. This would, therefore, extend the current interest in prestimulus brain oscillations.

How could the properties of alpha and theta waves and their changes be understood from the perspective of their possible roles in brain functions? Certainly, normal brain functions require dynamic interactions of functionally specialized but widely distributed cortical regions. Propagating waves have been proposed to coordinate these interactions by transferring or communicating information between different parts of large-scale neural networks and processing information based on their interactions [47]. In line with this proposal, waves propagating from frontal to occipital areas might be involved in signaling from association areas to sensory areas, which would imply top-down information flow. Likewise, waves propagating in an opposite direction may indicate a bottom-up information transfer. Hence, our analysis that prestimulus alpha waves traveling in the frontal to occipital direction were significantly correlated with responses may suggest the following scenario: the more top-down related information, such as expectancy and attention, was transferred, the faster subjects responded. This argument of the F-to-O alpha traveling waves in mediating top-down process is also supported by a previous animal study reporting that a phase shift of alpha oscillations from the upper level to the lower level of cortical hierarchy was related to a top-down-directed attention towards a relevant stimulus [48]. Our results, therefore, suggest that propagating waves play an important role for widely distributed brain areas to communicate with each other.
